# Virtual Reality Technology for Physical and Cognitive Function Rehabilitation in People With Multiple Sclerosis

**DOI:** 10.1155/2024/2020263

**Published:** 2024-09-24

**Authors:** MahgolZahra Kamari, Vitor Siqueira, Jemimah Bakare, Emerson Sebastião

**Affiliations:** ^1^ Graduate School of Comprehensive Human Sciences University of Tsukuba, 1-1-1 Tennodai, Tsukuba, Ibaraki 305-8577, Japan; ^2^ Department of Kinesiology and Physical Education Northern Illinois University, 1425 Lincoln Highway, DeKalb, Illinois, USA; ^3^ Department of Health and Kinesiology University of Illinois Urbana-Champaign, 906 South Goodwin Avenue, Urbana, Illinois 61801, USA

**Keywords:** cognition, dual task, mobility function, neurological disease, therapy

## Abstract

Virtual reality (VR) has significantly expanded the possibilities of medical treatment, particularly in the realm of rehabilitation. VR utilizes advanced technologies to create simulated environments that users perceive as analogous to the real world. Researchers have demonstrated that VR can effectively train motor, sensory, and cognitive functions. This manuscript offers a concise review of recent evidence concerning the effects of VR training on key clinical symptoms in people with multiple sclerosis (pwMS), with the aim of highlighting VR's potential as a complementary rehabilitative tool for improving ubiquitous symptoms of multiple sclerosis (MS)—a neurodegenerative, often disabling, disease. In addition to presenting a brief overview of recent literature on VR for pwMS, this narrative review seeks to provide health professionals with a foundational understanding of VR as a rehabilitative tool in MS. Furthermore, it may aid in identifying gaps in the literature and stimulate the development of new hypotheses and theories regarding the use of VR in patients with a neurodegenerative disease.

## 1. Introduction

Multiple sclerosis (MS) is a chronic, inflammatory, immune-mediated, and often disabling disease of the central nervous system (CNS) [1–3]. Recent reports show that MS affects around 2.8 million adults worldwide and over one million in the United States [3]. As one of the most common causes of neurological progressive disease, MS can lead to severe physical and cognitive impairment, including but not limited to mobility and cognitive impairment, balance and gait problems, muscle weakness, and sensory difficulties [4, 5]. These symptoms can impact an individual's ability to carry out daily activities, leading to a loss of independence and decreased participation in social, recreational, and vocational activities, ultimately affecting their quality of life. Despite advancements in understanding and treating MS over the past two decades, the disease remains incurable [6]. Therefore, the management of symptoms, restoration of function, and improvement of quality of life remain paramount for health professionals and clinicians involved in MS care [7, 8].

Rehabilitation, delivered by a multidisciplinary team of healthcare professionals including neurologists, general practitioners, MS nurses, and rehabilitation professionals such as neuropsychologists, psychologists, physiotherapists, occupational therapists, massage therapists, and exercise specialists, plays a crucial role in enabling individuals to live with MS. Rehabilitation is aimed at restoring function and teaching new strategies for carrying out daily tasks, thereby helping individuals maintain independence and self-empowerment [9]. To this end, rehabilitation programs adopt an integrated and comprehensive approach, utilizing a combination of individual or combined therapies and treatments to maximize the individual's overall health and well-being [10, 11]. Traditional physical and cognitive rehabilitation approaches for people with multiple sclerosis (pwMS) include onsite physical therapy, occupational therapy, supervised exercise training, memory exercises, problem-solving games, and mental exercises [12–14]. However, with advancements in technology, healthcare, and rehabilitation professionals, researchers now have access to a wide range of tools to better serve those living with MS. One such technological tool gaining traction in rehabilitation research is virtual reality (VR) [15].

VR presents an opportunity to deliver high-intensity, task-oriented, multisensory feedback training that can be easily adapted for home use [16]. This minimizes the need for onsite, supervised sessions and serves as a potential complement to primary rehabilitation treatments. Researchers have shown that VR can effectively train motor, sensory, and cognitive functions [16, 17]. Collectively, these findings highlight VR as a promising adjunctive tool for rehabilitating the common symptoms of MS, particularly physical and cognitive impairment. Moreover, VR has been suggested as a more motivating and cost-effective alternative to standard care [9, 18].

Given VR's potential as a rehabilitation approach to address prevalent MS symptoms, the goal of this narrative review is to underscore VR as an adjunctive rehabilitation strategy for improving the physical and cognitive function of pwMS. The general strategy for the presentation of this narrative review consists of four sections, beginning with this introduction, followed by a succinct discussion on the distinction between VR and augmented reality (AR). Subsequent sections explore the feasibility of VR for pwMS, its potential to enhance physical and cognitive function, and the challenges associated with its use in this population.

## 2. VR Versus AR

As we advocate for the integration of VR as a complementary rehabilitation approach, it is essential to differentiate VR from other similar existing technologies such as AR. At its core, VR relies on headsets, whereas AR may not necessarily require them. However, the differences between the two are far more complex.

The concept of VR has evolved over decades, with its origins traceable to the early 1960s when major airline companies and the air force introduced the first digital flight simulators [17]. The term “VR” was coined by Jaron Lanier in 1986 to describe a suite of technological devices, including a computer capable of interactive 3-dimensional visualization, a head-mounted display, and controllers equipped with one or more position trackers [18]. The medical field claims to be one of the most effective applications of VR [19]. VR, fundamentally, offers nearly authentic and believable experiences in a synthetic or virtual environment, achieved through advanced technologies [13, 15].

VR is characterized by three distinct features: interaction, immersion, and imagination [20]. Interaction refers to the natural engagement between the user and the virtual environment, providing a sense of realism through feedback. Immersion entails the sensation of being fully engaged in the virtual world, akin to being submerged in it. Imagination involves leveraging multidimensional perception information from VR scenes to evoke sensations akin to the real world while also eliciting novel experiences not possible in reality. VR can manifest in various accessible forms, including gaming consoles like Nintendo Wii, X-box Kinect, and controllers like Leap Motion, and can even extend to 360 immersive experiences like Oculus 360 [18]. These enriched environments offer personalized experiences, with opportunities for task repetition and feedback loops crucial for motor learning and skill acquisition. Importantly, all mentioned VR devices are readily available commercially.

In contrast, AR enriches the real world by overlaying computer-generated information onto it. It represents another innovative technological system that enhances sensory experiences of the physical environment by integrating virtual elements into the real-world view, typically through cameras, smartphones, or other vision devices [13, 21]. Unlike VR, the AR environment combines real and virtual objects within a physical environment [10].

## 3. VR and MS: Safety and Feasibility

Feasibility studies in rehabilitation are crucial in assessing the viability, safety, and potential of interventions, while also identifying risks and barriers. Recent investigations have indicated that VR interventions are not only feasible but also safe and well-tolerated for pwMS [22, 23] **(**Table 1**)**. For instance, one study explored the feasibility and tolerability of repeated VR sessions among pwMS (mean age: 50.1 ± 10.6 years), with a secondary objective of examining changes in positive and negative effects post-VR sessions [22]. The study achieved its feasibility target, with 100% of participants completing at least four out of eight intervention sessions, totaling 93% completion across all sessions. High levels of satisfaction with the VR experience were reported in a participant exit survey, with all individuals expressing willingness to engage in VR again [22].

The above is consistent with a previous study where pwMS reported greater enjoyment and commitment to therapy when receiving VR interventions compared to standard therapy alone [23]. Participants described the VR training sessions as helpful, challenging, enjoyable, and easy to comprehend. The authors noted a high willingness among participants to repeat the sessions, with 87% finding the training suitable. Moreover, the utilization of XRHealth software and the Oculus Rift Station platform was deemed feasible, safe, and engaging for patients [23].

Taken together, the results of the described studies strongly suggest that VR training therapy is not only feasible and safe but also engaging for pwMS.

## 4. Potential of VR to Improve Physical Function in MS

Physical function is defined as the ability to carry out both basic and instrumental activities of daily living [24]. The execution of motor tasks inherent in daily activities necessitates the intricate coordination of various physiological systems, including neuromotor, musculoskeletal, balance, gait, and cardiorespiratory systems [25]. Evidence suggests that pwMS commonly experience diminished physical function and encounter challenges with activities of daily living, alongside concerns regarding ongoing functional decline and mobility issues over time [3, 26]. By contrast, a recent study by Castellano-Aguilera et al. [27] demonstrated that VR offers real-time, multisensory feedback during motor training, enabling patients to enhance their performance in activities of daily living by acquiring new motor strategies. This section briefly summarizes recent research evaluating the efficacy of VR interventions in addressing specific MS-related symptoms related to physical function, such as balance, fatigue, walking ability, and mobility.  Table 2 summarizes the selected studies included.

### 4.1. VR Combined With Exergame

This describes the integration of exergames within a VR environment, a trend known as VR fitness or exergaming. Exergames, also referred to as active video games, utilize technology capable of tracking body movements, thereby transforming video gaming into a form of exercise and rehabilitation [29, 30]. Combining exergames with VR offers users an immersive and enhanced experience, providing realistic feedback and scenarios.

VR immersion in exergames is facilitated by VR sensors detecting body movements, synchronizing virtual and physical movements, thereby enhancing the suitability of VR for exergames [1, 28]. The interactive nature of exergames fosters user engagement, making VR fitness an ideal fusion of technology and exercise. This approach not only alleviates the monotony often associated with rehabilitation but also boosts patient motivation, offers direct feedback, and facilitates dual-task training. Several studies have explored the effects of exergames combined with VR on pwMS, yielding promising results: (a) improved balance and physical function [16] and (b) increased motivation and adherence [24, 29] and dual-task training [11, 31, 32]. For instance, García-Muñoz et al. [28] conducted a case study involving a 54-year-old woman with relapsing-remitting MS, implementing an immersive VR vestibular training protocol. Following the VR intervention (up to 20 sessions, 50 min per session, and it was performed 2-3 times per week for 7 weeks), improvements were observed across various parameters, including balance, gait, fatigue impact, and muscular tone. The authors concluded that the VR exergame intervention holds promise for enhancing mobility and postural control in pwMS.

Additional studies have evaluated the efficacy of VR with exergame on physical function aspects of pwMS. For instance, Nascimento et al. [3] analyzed the efficacy of exergaming and VR for balance recovery, and physical function in MS patients was assessed. Comparing VR with conventional interventions, the authors found VR training to be equally effective in improving physical function in pwMS. Furthermore, another systematic review with meta-analysis evaluated the efficacy of exergaming and VR for balance recovery in pwMS. The analysis demonstrated a significant improvement in the Berg Balance Scale, with VR exergames showing a significant effect size compared to conventional rehabilitation [27]. Although further research is warranted to delineate VR's specific role in neurological rehabilitation, existing evidence underscores the positive impact of VR-based training on physical function and postural control in pwMS. Overall, rehabilitative interventions incorporating VR appear to offer greater effectiveness than conventional approaches in improving physical function in pwMS.

### 4.2. VR in Rehabilitation Therapy Interventions

VR technology has become increasingly integrated into rehabilitation and therapeutic interventions for individuals with neurological diseases, offering multiple sensory inputs such as visual, auditory, and haptic feedback to create immersive and enriched rehabilitation experiences. In the context of MS, VR has shown promise in reducing fatigue, improving balance, and enhancing the overall quality of life for patients.

A recent systematic review with a meta-analysis conducted by Cortés-Pérez et al. [25] examined the efficacy of VR in reducing fatigue, mitigating the impact of MS, and enhancing the overall quality of life in pwMS. This comprehensive review included randomized controlled trials involving individuals with MS who received VR-based therapy compared to conventional therapy (CT) or simple observation. Assessment outcomes encompassed fatigue, MS impact, and quality of life. The analysis, based on data from 12 trials involving 606 individuals with MS, revealed that VR-based therapy was superior to CT in reducing fatigue and improving the mental dimension of quality of life. Additionally, VR-based therapy outperformed simple observation in reducing the impact of MS and enhancing overall quality of life. When combined with CT, VR demonstrated greater effectiveness than CT alone in improving various dimensions of quality of life.

Similarly, Wallin et al. [24] conducted a systematic review with a meta-analysis of randomized controlled trials evaluating the efficacy of VR interventions in individuals with MS compared to control training or simple observation. The review, encompassing 26 studies, revealed that VR interventions ranging from 4 weeks to 4 months in duration, with sessions occurring one to five times per week and lasting 20–60 min per session, effectively reduced the disabling impact of MS symptoms. Their findings highlighted the efficacy of VR interventions in reducing fatigue, mitigating the impact of MS, and enhancing overall quality of life in pwMS.

## 5. Potential of VR to Improve Cognitive Function in MS

Many activities of daily living involve multitasking and require significant cognitive engagement. Cognitive impairment is a common symptom of MS, affecting approximately 40%–70% of pwMS [28]. Among the most prevalent cognitive deficits observed in MS are slowed information processing speed and impairments in learning and memory [33]. However, challenges in other cognitive domains, including attention, working memory, executive functions, visuospatial processing, social cognition, and certain aspects of language (e.g., verbal fluency), may also manifest [33]. These cognitive impairments profoundly impact overall well-being, interfering with both occupational and social functioning [34]. Moreover, cognitive decline in pwMS often occurs early in the course of the disease and can detrimentally affect mobility during daily activities [25, 28, 34]. Even pwMS who appear physically and/or cognitively preserved may experience difficulties in simultaneously performing cognitive tasks and walking, a phenomenon known as dual-task interference [28]. From this perspective, VR holds significant promise in (neuro)rehabilitation.

Research studies suggest that VR can enhance the effectiveness of cognitive rehabilitation and exercise training in pwMS by augmenting sensory input and promoting multisensory processing [33, 34]. VR provides a dynamic and interactive therapy experience with instant feedback, which can enhance patient motivation and adherence to therapy sessions [35, 36]. While cognitive rehabilitation and exercise training represent promising behavioral approaches to address cognitive deficits in pwMS, their effects are often modest and may not consistently translate into improvements in everyday functioning [37]. However, VR has the potential to amplify the outcomes of cognitive rehabilitation and exercise training by bolstering sensory input and facilitating multisensory integration and processing during rehabilitation sessions [28, 34]. This section briefly summarizes studies that evaluated the effectiveness of VR on cognitive function in pwMS.  Table 3 displays detailed information on the selected included studies.

### 5.1. VR Combined With Exergame

A systematic review conducted by Moeinzadeh et al. [38] compared the effects of conventional exercise and VR exergaming on the cognitive abilities of pwMS. Various gaming platforms and conventional exercise regimens were utilized across the selected studies (comprising 39 articles, of which 10 met the eligibility criteria), with measures of cognitive abilities employed to assess the impact of VR exergaming relative to conventional exercise. The review revealed a positive influence of VR exergaming on MS rehabilitation, with VR exergaming generally outperforming conventional exercise in improving cognitive abilities and information processing. By combining exergames with VR, patients' CT progress in cognitive functions can be integrated, offering a daily-life environment conducive to cognitive-motor training improvement. VR exergaming, thus, emerges as a promising training approach to enhance cognitive functions, particularly brain–body communication, in pwMS [17, 38–40].

### 5.2. VR in Rehabilitation Therapy Interventions

A recent meta-analysis conducted by Wender et al. [32] proposed a conceptual framework supporting the integration of VR as a common and ideal adjuvant therapy to traditional cognitive rehabilitation and exercise training in pwMS. Involving 240 pwMS, the study highlighted VR as a compelling candidate for augmenting conventional cognitive rehabilitation and exercise training due to its stimulating, engaging, complex, and ecologically valid nature. The combination of VR with traditional approaches was found to facilitate enhanced cognitive improvements compared to standalone cognitive rehabilitation or exercise training methods. Furthermore, such combined interventions were deemed more likely to lead to long-term improvements in everyday functioning. The authors emphasized that integrating multisensory feedback and integration into cognitive rehabilitation and exercise training through VR could yield more substantial cognitive improvements. Galperin et al. [39] found that a 6-week treadmill training program combined with VR resulted in greater improvements in dual-tasking gait speed and cognitive processing speed compared to treadmill training alone, with these effects sustained at a 3-month follow-up. Leonardi et al. [40] demonstrated that an 8-week VR-based cognitive rehabilitation program significantly improved executive and visuospatial functions in pwMS, suggesting that the VR approach can lead to substantial cognitive enhancements.

Similarly, a recent systematic review examined the role of VR in cognitive rehabilitation among pwMS, revealing positive effects on various cognitive deficits. The findings suggested significant enhancements across multiple cognitive domains, including executive and visual-spatial abilities, speech, attention, and memory skills, following VR training [28]. This multisensory integration inherent in VR interventions holds the potential to enhance motor learning, cognitive processing, and the transfer of skills to daily life activities.

## 6. Potential Challenges Associated With the use of VR in pwMS

Together with the potential physical and cognitive benefits for the use of VR as a rehabilitation tool for pwMS, exposure to immersive VR may also be associated with potential problems and challenges in this population that include but are not limited to physical and cognitive challenges, technological and accessibility challenges, safety concerns, and psychological barriers.

### 6.1. Physical and Cognitive Challenges

pwMS may experience physical limitations, such as reduced mobility, balance problems, and fatigue [15, 32], which may make it challenging to interact with and navigate certain VR environment. Cognitive deficits common in MS, such as impaired information processing speed, attention, memory, and executive function [14] may also hinder the effective use of VR technologies. To this end, careful consideration is warranted to ensure that VR tasks and environments are appropriately designed and adapted to the individual's physical and cognitive capabilities.

### 6.2. Technological and Accessibility Challenges

Access to high-quality VR hardware and software can be limited, especially for pwMS with lower socioeconomic status or in resource-constrained settings. This is reinforced by a recent work addressing social determinants of health in MS [41]. Technical issues such as motion sickness, delays in visual and auditory feedback, and compatibility problems can reduce the usability and acceptability of VR systems. Lack of technical support and training for both patients and clinicians may pose barriers to the effective implementation of VR in rehabilitation.

### 6.3. Safety

There may be an increased risk of falling or getting injured due to the immersive nature of VR and possible balance and mobility issues in pwMS. Falls are highly prevalent in pwMS and can result in negative consequences such as injury, activity curtailment, reduced quality of life, and increased need for care and time off work [42]. Prolonged exposure to VR may lead to increased symptoms such as transient dizziness, nausea, disorientation, and impaired postural control [43]. Therefore, it is essential to have careful screening, monitoring, and safety protocols to reduce such risks.

### 6.4. Psychological Barriers

Some individuals, particularly older adults or those with limited technological experience, may feel hesitant or anxious about using new technology. There is some evidence suggesting that age and level of education are important factors associated with the acceptability of technology in health care among older adults [44]. With the shifting in peak prevalence in MS [24, 44], this warrants attention. Moreover, such feelings can be especially true when it comes to VR, where concerns about cybersickness, feeling overwhelmed, or a loss of control in the virtual environment may arise. Therefore, it is important to provide adequate training, familiarization, and emotional support to assist pwMS feel comfortable and confident while using VR.

## 7. Final Consideration

This narrative is aimed at underscoring VR as an adjuvant rehabilitation approach to improve physical and cognitive function in pwMS. There is a high prevalence of physical and cognitive impairment in pwMS and the development of new strategies to help restore function in this population is of great importance, particularly strategies that reduce the burden of and increase motivation of patients. Recent evidence suggests that VR has the potential to improve physical and cognitive function in pwMS. This narrative summarized recent studies that collectively demonstrated that VR provides the opportunity to expand the accessibility of both physical and cognitive rehabilitation services and offers a potential ally to help improve ubiquitous symptoms of MS, namely, physical and cognitive function, with the potential of enhancing adherence to rehabilitation programs. Here, we described studies that collectively demonstrated that VR represents a strong candidate as a stimulating, engaging, complex, and ecologically valid addition to conventional rehabilitation approaches for managing MS-related physical and cognitive impairment. However, it is important to understand that there may be potential challenges associated with the use of VR in pwMS (i.e., physical and cognitive challenges, technological and accessibility challenges, safety concerns, and psychological barriers).

Despite the described challenges, VR has several advantages when compared to traditional rehabilitation approaches. First, VR has a high level of ecological validity because of the sensorimotor interaction between the user and the virtual environment, allowing to transfer of skills from the virtual to the real world. Second, the compliance and satisfaction of the patient when interacting with the enriched computer-generated environment. Third, VR has the great advantage of providing immediate and direct feedback. Collectively, the aforementioned underscores VR as a rehabilitative tool that can be used and easily implemented alongside mainstream physical and cognitive rehabilitation programs to improve important clinical and prevalent symptoms of MS.

In conclusion, VR has been used in the rehabilitation of several neurological diseases including pwMS to enhance both physical and cognitive function as it offers task-oriented exercises enhancing motor learning and neural plasticity. The present narrative review provides evidence that the use of VR rehabilitation is a feasible, safe, and potentially effective approach for improving both physical and cognitive function in pwMS. VR seems to be a promising strategy for enhancing different components of physical function such as balance, and mobility, and an adjuvant to traditional cognitive rehabilitation and exercise training for managing cognitive impairment in this population [45]. However, continued research is warranted to further elucidate the optimal implementation of VR interventions and their long-term effects on the holistic well-being of pwMS.

## Figures and Tables

**Table 1:** Summary of the feasibility studies of virtual reality in people with multiple sclerosis.

**Study**	**Sample**	**Intervention length**	**Type of VR equipment and application**	**VR protocol**	**Primary outcome**	**Secondary outcome**	**Main findings**
Shaw et al. (2021) [22]	16 pwMS	4 weeks	HTC Vive VR platform Karuna Lab's Virtual Embodiment Training, which is designed to relieve pain via physical interaction and is based on physical therapy and cognitive neuroscience.	Eight VR sessions were administered over 4 weeks	Feasibility and tolerability	Change in effect	VR interventions are feasible, safe, and tolerable for individuals living with MS and may improve effect.

Kalron et al. (2022) [23]	26 pwMS	Single 45-min digital environment session with VR	Oculus Rift and XRHealth software XRHealth applications in the form of games and exercises that focus on motor, cognitive, and mental experiences		Feasibility	NA	XRHealth software and the Oculus Rift Station platform are feasible, safe, and engaging for pwMS.

Abbreviations: NA, not applicable; PwMS, people with multiple sclerosis; VR, virtual reality.

**Table 2:** Summary of the selected studies evaluating the effects of virtual reality interventions on physical function in people with multiple sclerosis.

**Study**	**Sample**	**Intervention length**	**Type of VR equipment and application**	**VR protocol**	**Primary outcome**	**Secondary outcome**	**Main findings**
García-Muñoz et al. (2022) [28]	One case	7 weeks, 2–3 sessions/week	The Oculus Quest, a wireless VRi, applies an Oculus Quest, a wireless VRi vestibular training protocol, and assesses the impact of fatigue, and quality of life by the experimental intervention.	Up to 20 sessions, 50 min/session	Assess the balance, fatigue, and QoL	Muscle Tone 2 evaluations out of 4 were carried out. Postintervention and 1 month after the experimental procedure	Improvement in balance, gait, fatigue, and muscle tone.
Cortés-Pérez et al. (2021) [25]	606 PwMS	4 weeks to 4 months, 1–5 sessions/20–60 min	A systematic review with meta-analysis RCTs with PwMS that received VR-based therapy in comparison to CT.	Meta-analysis was conducted through a bibliographic search on PubMed, Scopus, Web of Science, and PEDro up to April 2021	Fatigue, MS impact, QoL	NA	VRBT is effective at reducing fatigue and MS impact and improving QoL.
Wallin et al. (2019) [24]	Evaluating randomized controlled trials	4 weeks to 4 months, 1–5 sessions	Assessed the epidemiology of MS from 1990 to 2016.	MS were modeled with DisMod-MR version 2.1, a Bayesian meta-regression framework widely used in GBD epidemiological modeling.	The effect of VR-based therapy on fatigue, QoL, and fatigue	NA	VR-based therapy is effective at reducing fatigue and increasing quality of life.
Nascimento et al. (2021) [3]	209 PwMS	Nine randomized clinical trials with a total sample of 424 participant	Systematic review and meta-analysis of randomized controlled trials.	Assess an exergames -VR vs. conventional rehabilitation	the positive effect of using VR in people with MS in relation to fatigue, QoL, and balance, compared to the conventional exercises.	NA	Exergames had a significant effect on balance, fatigue, and OoL
Ozdogar et al. (2020) [29]	Three groups: video-based exergaming (*n* = 21), conventional rehabilitation (*n* = 19), and control groups (*n* = 20)	8 weeks	The video-based exergaming used a game console (Microsoft Xbox One and Kinect motion sensor), conventional rehabilitation, and no intervention control.	Once per week for 8 weeks	The primary outcome was the nine-hole peg test (N-HPT], a valid and widely used tool to measure dexterity in PwMS	NA	Most of the function and balance-related outcomes were significantly improved
Khalil et al. (2018) [11]	Intervention group) (*n* = 16) and a control group (*n* = 16)	6 weeks	In the nonimmersive, low-cost system, a large standard LCD monitor was used.	CG = At home balance exercises three times per week for 6 weeks. VRG =VR-based balance exercises two times per week for 6 weeks + the same as the CG.	VR Scenario measured the participants' balance through the Berg Balance Scale	Gait, QoL, fatigue, and functional capacity were measured	The study showed significant improvement in fatigue and QoL

Abbreviations: CG, control group; CT, conventional therapy; GBD, global burden of diseases; LCD, liquid crystal display; NA: not applicable; QoL, quality of life; RCT, randomized controlled trials; VR, virtual reality; VRG, virtual reality group; VRi, immersive virtual reality.

**Table 3:** Summary of selected studies evaluating the effects of virtual reality interventions on cognitive function in people with multiple sclerosis.

**Study**	**Sample**	**Intervention length**	**Type of VR equipment and application**	**VR protocol**	**Primary outcome**	**Secondary outcome**	**Main findings**
Wender et al. (2022) [32]	240 PwMS	NA	VR and a conceptual framework supporting the use of VR as an ideal, common adjuvant traditional CR and ET in MS.	VR as an adjunct to conventional cognitive rehabilitation and exercise training.	VR could strengthen the effects of CR and ET by increasing sensory input	VR improves everyday function	VR is a strong candidate as a stimulating, engaging cognitive improvement
Moeinzadeh et al. (2023) [38]	10 studies were considered	NA	Various gaming platforms and conventional exercises were used based on the extent of technologies and therapy regimens.	Exergames or VR vs. conventional exercise	Positive impacts of VR-exergaming in MS rehabilitation	NA	VR exergames were more effective than traditional exercises in improving cognitive abilities.
Maggio et al. (2019) [35]	28 studies were considered	Studies published from 2007-2018	Searching on Scopus, PubMed, Web of Science, and Cochrane database all the studies fulfilling our selected criteria and published between 2007 and 2018	28 articles used to select motor and cognitive studies in patients with MS	Cognitive and motor outcomes	Long-term visuospatial memory, Motor impairment, and depression were measured	VR has positive effects on various cognitive domains in PwMS.
Abd-Alrazaq et al. (2022) [34]	A systematic review of randomized controlled trials was conducted	12 weeks (August 2021–November 2021)	They consulted two experts in digital mental health and checked the search query used in other systematic reviews within this field.	The chosen search terms related to the target population (cognitive impairment), target intervention (serious games and exergames), and target study design (RCT and clinical trial)	Improvement of Cognitive abilities in elderly with cognitive impairment	NA	Serious games with VR were effective in improving cognitive abilities in the elderly with cognitive impairment.
Galperin et al., 2022 [39]	*N* = 108	6 weeks and following up measurement after 3 months	Single-blinded, 2-arm RCT that took place at four clinical sites. Participants were randomized to either treadmill training with VR (TT + VR) or TT alone group. Symbol digit modalities test (SDMT) score.	3/week × 6 weeks were applied. VR was projected on the TV screen in front of the participants in the TT + VR Group.	Dual-tasking gait speed and cognitive processing speed improved	Measures included functional gait tests and, cognitive function.	The impact on cognitive processing speed was greater after TT + VR training than after TT (*p* < 0.008).
Leonardi et al., 2021 [40]	*N* = 30	8 weeks	Rehabilitation training with the VR rehabilitation system.	Subjects received CR, 3 times a week × 8 weeks (each session lasting about 45 min).	The cognitive rehabilitative program, including the standard and the experimental (VRRS)	Executive and visuospatial functions were measured	Conventional and VR cognitive rehabilitation approaches improved

Abbreviations: CR, cognitive rehabilitation; ET, exercise training; NA, not applicable; PwMS, people with multiple sclerosis; RTC, randomized control trials; TT, treadmill training; VRRS, virtual reality rehabilitation system; VR, virtual reality.

## Data Availability

This study is a narrative review, and as such, no new datasets were generated or analyzed during this study. The underlying data supporting the results can be found in the primary sources referenced throughout the manuscript. Where applicable, links to publicly accessible articles and datasets are provided in the reference list. Access to some materials may be restricted based on journal access policies or subscription requirements.

## References

[1] Sagawa Y., Watelain E., Moulin T., Decavel P. (2021). Physical activity during weekdays and weekends in persons with multiple sclerosis. *Multiple Sclerosis Journal-Experimental, Translational and Clinical, Sensors*.

[2] Pagiari C., Tella S. D., Johanna J. (2021). Effects of home-based virtual reality telerehabilitation system in people with multiple sclerosis: a randomized controlled1 trial. *Journal of Telemedicine and Telecare*.

[3] Nascimento A. S., Fagundes C. V., Mendes F. A. D. S., Leal J. C. (2021). Effectiveness of virtual reality rehabilitation in persons with multiple sclerosis: a systematic review and meta-analysis of randomized controlled trials. *Multiple Sclerosis and Related Disorders*.

[4] Cree B. A. C., Oksenberg J. R., Hauser S. L. (2022). Multiple sclerosis: two decades of progress. *Journal of NeuroEngineering and Rehabilitation*.

[5] Chan L. P. P., Cheng Y., Ng J. Y. H., Zheng Z., Cheing G. L. Y. (2022). A review of virtual reality technology in exercise training for older adults. *Journal of Endocrinology and Thyroid Research*.

[6] Libak A., Aditya A., Alexander F., Yiting D., Laura A. (2021). Effectiveness of physical therapy interventions in reducing fear of falling among individuals with neurologic diseases: a systematic review and meta-analysis. *Archives of Physical Medicine and Rehabilitation*.

[7] Hakan A., Gülce K. S., Burak A., Erdem K. (2022). The effect of virtual reality-based therapy on fear of falling in multiple sclerosis: a systematic review and meta-analysis. *Multiple Sclerosis and Related Disorders*.

[8] Yeroushalmi S., Maloni H., Costello K., Wallin M. T. (2019). Telemedicine and multiple sclerosis: a comprehensive literature review. *Journal of Telemedicine and Telecare*.

[9] Khan F., Amatya B., Kesselring J., Galea M., Cochrane Multiple Sclerosis and Rare Diseases of the CNS Group (2015). Telerehabilitation for persons with multiple sclerosis. *Cochrane Database of Systematic Reviews*.

[10] Wang S., Li H. Y. G., Jia Y. (2020). Detection of mild cognitive impairment based on virtual reality: a scoping review. *Journal of Bentham Science*.

[11] Khalil H., Al-Sharman A., El-Salem K. (2019). The development and pilot evaluation of virtual reality balance scenarios in people with multiple sclerosis (MS): a feasibility study. *Journal of Neurorehabilitation*.

[12] Molhemi F., Monjezi S., Mehravar M. (2021). Effects of virtual reality vs conventional balance training on balance and falls in people with multiple sclerosis: a randomized controlled trial. *Archives of Physical Medicine and Rehabilitation*.

[13] Foley F. W., Portnoy J. G. (2018). Neuropsychology in the integrated MS care setting. *Archives of Clinical Neuropsychology*.

[14] Tacchino A., Podda J., Bergamaschi V., Pedullà L., Brichetto G. (2023). Cognitive rehabilitation in multiple sclerosis: three digital ingredients to address current and future priorities. *Frontiers in Human Neuroscience*.

[15] Invernizzi C. M., Ammendolia M., Marotta N. (2021). Efficacy of Virtual Reality and Exergaming in Improving Balance in Patients With Multiple Sclerosis. *Neurorehabilitation*.

[16] Philip D. (2022). The analgesic effects of virtual reality for people with chronic pain: a scoping review. *Pain Medicine*.

[17] Schättin A., Häfliger S., Meyer A. (2021). Design and evaluation of user-centered exergames for patients with multiple sclerosis: multilevel usability and feasibility studies. *Journal of Medical Internet Research*.

[18] Wan-Yu H., Anguera J. A., Rizzo A. (2023). A virtual reality program to assess cognitive function in multiple sclerosis: a pilot study. *Frontiers in Human Neuroscience*.

[19] Pantelidis V. S. (2010). Reasons to use virtual reality in education and training courses and a model to determine when to use virtual reality. *Themes in Science and Technology Education*.

[20] Kim Y., Kim H., Kim Y. O. (2017). Virtual reality and augmented reality in plastic surgery: a review. *Archives of Plastic Surgery*.

[21] Yang L. I., Huang J., Tian F., Wang H., Dai G. Z. (2019). Gesture interaction in virtual reality. *Virtual Reality & Intelligent Hardware*.

[22] Shaw M. T., Palmeri M. J., Malik M., Dobbs B., Charvet L. E. (2021). Virtual reality is a feasible intervention platform in multiple sclerosis: a pilot protocol and acute improvements in affect. *Multiple Sclerosis Journal, Experimental, Translational and Clinical*.

[23] Kalron A., Frid L., Fonkatz I. (2022). The design, development, and testing of a virtual reality device for upper limb training in people with multiple sclerosis: single-center feasibility study. *Journal of Medical Internet Research*.

[24] Wallin M. T., Culpepper W. J., Nichols E. (2019). Global, regional, and national burden of multiple sclerosis 1990–2016: a systematic analysis for the global burden of disease study 2016. *Lancet Neurology*.

[25] Cortés-Pérez I., Sánchez-Alcalá M., Nieto-Escámez F. A., Castellote-Caballero Y., Obrero-Gaitán E., Osuna-Pérez M. C. (2021). Virtual reality-based therapy improves fatigue, impact, and quality of life in patients with multiple sclerosis. a systematic review with a meta-analysis. *Sensors Basel*.

[26] Calafiore D., Invernizzi M., Ammendolia A. (2021). Efficacy of virtual reality and exergaming in improving balance in patients with multiple sclerosis: a systematic review and meta-analysis. *Frontiers in Neurology*.

[27] Castellano-Aguilera A., Biviá-Roig G., Cuenca-Martínez F. (2022). Effectiveness of virtual reality on balance and risk of falls in people with multiple sclerosis: a systematic review and meta-analysis. *International Journal of Environmental Research and Public Health*.

[28] García-Muñoz C., Cortés-Vega M. D., Hernández-Rodríguez J. C., Fernández-Seguín L. M., Escobio-Prieto I., Casuso-Holgado M. J. (2022). Immersive virtual reality and vestibular rehabilitation in multiple sclerosis: case report. *Journal of Medical Internet Research*.

[29] Ozdogar A. T., Ertekin O., Kahraman T., Yigit P., Ozakbas S. (2020). Effect of video-based exergaming on arm and cognitive function in persons with multiple sclerosis: a randomized controlled trial. *Multiple Sclerosis and Related Disorders*.

[30] Sanai S. A., Saini V., Benedict R. H. B. (2016). Aging and multiple sclerosis. *Journal of Multiple Sclerosis*.

[31] Sandroff B. M., DeLuca J. (2020). Will behavioral treatments for cognitive impairment in multiple sclerosis become standards-of-care?. *International Journal of Psychophysiology*.

[32] Wender C. L. A., DeLuca J., Sandroff B. M. (2022). Developing the rationale for including virtual reality in cognitive rehabilitation and exercise training approaches for managing cognitive dysfunction in MS. *Journal of Neuroscience*.

[33] Benzing V., Schmidt M. (2018). Exergaming for children and adolescents: strengths, weaknesses, opportunities, and threats. *Journal of Clinical Medicine*.

[34] Abd-Alrazaq A., Alajlani M., Alhuwail D. (2022). The effectiveness and safety of serious games for improving cognitive abilities among elderly people with cognitive impairment: systematic review and meta-analysis. *Journal of Medical Internet Research*.

[35] Maggio M. G., Russo M., Cuzzola M. F. (2019). Virtual reality in multiple sclerosis rehabilitation: a review on cognitive and motor outcomes. *Journal of Clinical Neuroscience*.

[36] Taylor L. A., Mhizha-Murira J. R., Smith L. (2021). Memory rehabilitation for people with multiple sclerosis. *Cochrane Database of Systematic Reviews*.

[37] Dalmazane M., Gallou-Guyot M., Compagnat M. (2021). Effects on gait and balance of home-based active video game interventions in persons with multiple sclerosis: a systematic review. *Multiple Sclerosis and Related Disorders*.

[38] Moeinzadeh A. M., Calder A., Petersen C., Hoermann S., Daneshfar A. (2023). Comparing virtual reality exergaming with conventional exercise in rehabilitation of people with multiple sclerosis: a systematic review. *Neuropsychological Rehabilitation*.

[39] Galperin I., Mirelman A., Schmitz-Hübsch T. (2023). Treadmill training with virtual reality to enhance gait and cognitive function among people with multiple sclerosis: a randomized controlled trial. *Journal of Neurology*.

[40] Leonardi S., Maggio M., Russo M. (2021). Cognitive recovery in people with relapsing/remitting multiple sclerosis: a randomized clinical trial on virtual reality-based neurorehabilitation. *Clinical Neurology and Neurosurgery*.

[41] Dobson R., Rice D. R., D'hooghe M. (2022). Social determinants of health in multiple sclerosis. *Nature Reviews Neurology*.

[42] Coote S., Comber L., Quinn G., Santoyo-Medina C., Kalron A., Gunn H. (2020). Falls in people with multiple sclerosis: risk identification, intervention, and future directions. *International Journal of MS Care*.

[43] Pau M., Arippa F., Leban B. (2024). Cybersickness in people with multiple sclerosis exposed to immersive virtual reality. *Bioengineering*.

[44] Vaughn C. B., Jakimovski D., Kavak K. S. (2019). Epidemiology and treatment of multiple sclerosis in elderly populations. *Nature Reviews Neurology*.

[45] Baniasadi T., Ayyoubzadeh S. M., Mohammadzadeh N. (2020). Challenges and practical considerations in applying virtual reality in medical education and treatment. *Oman Medical Journal*.

